# Distinct fermentation and antibiotic sensitivity profiles exist in salmonellae of canine and human origin

**DOI:** 10.1186/s12866-018-1153-4

**Published:** 2018-02-26

**Authors:** Corrin V. Wallis, Preena Lowden, Zoe V. Marshall-Jones, Anthony C. Hilton

**Affiliations:** 1 The WALTHAM Centre for Pet Nutrition, Melton Mowbray, Leicestershire, LE14 4RT UK; 2 Life and Health Sciences, Aston University, B4 7ET, Birmingham, UK

**Keywords:** *S. Enterica*, Serovar, Dog, Zoonosis, Anthroponosis, Biolog, MIC

## Abstract

**Background:**

*Salmonella enterica* is a recognised cause of diarrhoea in dogs and humans, yet the potential for transfer of salmonellosis between dogs and their owners is unclear, with reported evidence both for and against *Salmonella* as a zoonotic pathogen. A collection of 174 *S. enterica* isolates from clinical infections in humans and dogs were analysed for serotype distribution, carbon source utilisation, chemical and antimicrobial sensitivity profiles. The aim of the study was to understand the degree of conservation in phenotypic characteristics of isolates across host species.

**Results:**

Serovar distribution across human and canine isolates demonstrated nine serovars common to both host species, 24 serovars present in only the canine collection and 39 solely represented within the human collection. Significant differences in carbon source utilisation profiles and ampicillin, amoxicillin and chloramphenicol sensitivity profiles were detected in isolates of human and canine origin. Differences between the human and canine *Salmonella* collections were suggestive of evolutionary separation, with canine isolates better able to utilise several simple sugars than their human counterparts. Generally higher minimum inhibitory concentrations of three broad-spectrum antimicrobials, commonly used in veterinary medicine, were also observed in canine *S. enterica* isolates.

**Conclusions:**

Differential carbon source utilisation and antimicrobial sensitivity profiles in pathogenic *Salmonella* isolated from humans and dogs are suggestive of distinct reservoirs of infection for these hosts. Although these findings do not preclude zoonotic or anthroponotic potential in salmonellae, the separation of carbon utilisation and antibiotic profiles with isolate source is indicative that infectious isolates are not part of a common reservoir shared frequently between these host species.

## Background

*Salmonella* is a common cause of food poisoning and a recognised intestinal pathogen in both humans and animals [1, 2]. The most common cause of infection in humans is through consumption of meat and dairy products, raw fruits and vegetables or through contact with environmental contamination [3–5]. Zoonotic transmission to humans from companion animals is also reported and continues to be considered a largely unquantified risk [6–8]. The understanding of this potential reservoir of infection is complicated by the carriage of *Salmonella* in dogs and cats without clinical signs [2, 9].

The prevalence of *Salmonella* carriage in clinically healthy dogs varies with geographical location, lifestyle and diet, and has been reported to range from 0 to 69% [2, 10–13]. Few recent studies of the prevalence of *Salmonella* in dogs from the UK exist, yet those conducted within recent decades suggest low carriage rates in this geographical population. Indeed two UK studies found only a single dog positive for *Salmonella* in cohorts of 253 [14] and 436 [12] subjects, while a recent multicentre study of carriage in dogs from the United States reported 2.5% of healthy dogs carried the organism, increasing to 3.8% in diarrhoeic pets [15]. Stray or shelter housed dogs and cats have been reported to pose a particular risk for human salmonellosis infection [16, 17]. However, carriage rates congruent with home owned pets have more recently been described within these populations [18]. Lifestyle-associated stress has similarly been suggested as a putative factor in increased carriage rates of salmonellae in dogs. Supporting data for this hypothesis was observed in a study of healthy pre-race and diarrheic racing Alaskan sled dogs with high *Salmonella* carriage rates of 69% (18/26) and 63% (19/30) detected respectively [19]. Feeding regime may however confound such hypotheses and could represent another important factor impacting carriage rates and clinical signs in companion animals [15, 20–24]. Joffe et al., [25] identified *Salmonella* in 30% of faeces samples from Canadian dogs receiving raw chicken diets, with the organism isolated from 80% of the diet samples. Published case studies describe clinical salmonellosis in cats apparently associated with the consumption of raw meat based diets [24, 26], while an outbreak of salmonellosis in US pet owners was directly attributed to contamination of raw pet food products [27]. A recent UK study also identified raw meat consumption as a risk factor for the carriage of clavulanate–amoxicillin resistant *Escherichia coli* in non-antimicrobial treated and non-veterinarian-visiting dogs [28]. Despite these observed cases and the increasing trend in feeding raw meat based diets however, carriage rates of *Salmonella* in pet animals is suggested to have declined in recent decades [15].

The socioeconomic costs associated with human salmonellosis have resulted in the organism being a major focus for The Foodborne Diseases Active Surveillance Network (FoodNet) over the past two decades [29]. The intelligence gained from such research represents an important factor in public health efforts to understand and control disease. Surveillance studies by FoodNet and others to identify trends in the incidence, sources and means of transfer of pathogenic salmonellae, are conducted by epidemiological analyses of confirmed cases, as well as through characterisation of isolates to understand relatedness and identify potential common sources or reservoirs [30, 31]. Studies to identify transmission routes, sources and reservoirs of infection have been conducted using a variety of standard microbiological, molecular and phenotypic methods including bacterial culture, serotyping, phage-typing, antibiotic resistance profiling, plasmid typing and whole genome sequence analyses [5, 32, 33]. To provide the level of granularity required for distinguishing isolate source, analyses involve detailed bacterial characterisation and frequently adopt a multifaceted approach combining methods such as serotyping, phage-typing, PCR, restriction fragment PFGE and antimicrobial sensitivity testing. More recently whole genome sequencing and the description of a stable, core genome has been described to show an enhanced ability to detect transmission routes and ecological relationships than the more plastic antimicrobial resistance profiles [34].

Whilst many serovars have been isolated from both animals and humans, salmonellae with a restricted host range have also been described [35, 36]. This host adaptation is typically characterised by an increased association of a particular serovar with a defined host. Examples of host adaptation include *Salmonella enterica* serovar Dublin, which is associated with cattle and *Salmonella enterica* subsp. *enterica* Serovar Typhimurium Variant Copenhagen Phage Type 99, associated with pigeons [36, 37]. Although cattle are considered the primary reservoir of *Salmonella* Dublin, which can persist sub-clinically within this host for long periods, interspecies transmission is observed into human and canine hosts. The acquisition of adaptive phenotypes may occur through horizontal gene transfer resulting in a rapid evolution of traits such as increased resistance to immune clearance [38, 39], or may occur through a gradual adaptive commensurate shift in metabolic capacity resulting in a competitive advantage. Host adaptation has not been reported in dogs, with studies of canine salmonellae identifying multiple serotypes shed in faeces [9]. However, since differences exist in the, albeit omnivorous, dietary intake in humans and dogs, the nutritional influences on salmonellae infecting these hosts may be distinct.

Metabolic profiling has been widely used in bacterial typing to determine species identification, evaluate microbial communities in food production and to inform diagnostic and intervention strategies for medically important bacterial pathogens [32, 40–43]. The development of systems such as the Biolog Gen III phenotypic array (Biolog, UK) allow simultaneous determination of multiple metabolic characteristics and have been applied to studies of plant and soil microbial ecology, food microbiology, wastewater contamination and bacterial species of clinical importance including *Salmonella* spp. [41, 43–48]. These methods may also be useful for the detailed characterisation of bacterial pathogens to determine commonalities and differences in their metabolic phenotypes.

This study sought to investigate salmonellae isolated from clinical diarrhoeal infections of humans and dogs to determine whether isolates from these hosts possess similar metabolic and phenotypic profiles. The null hypothesis for investigation was that *Salmonella* isolates from humans and dogs possess similar metabolic and phenotypic profiles. Acceptance of the null hypothesis would suggest a common pool of isolates with infections occurring through zoonotic and, or anthroponotic transfer between these hosts, while rejection of the null hypothesis may be indicative of metabolic shifts within distinct sub-populations and suggest separation of the reservoirs of infection.

## Methods

### Bacterial isolates and culture conditions

*Salmonella enterica* isolated from infections in human and canine patients with clinical diarrhoea were resurrected from cryogenic storage at − 80 °C by inoculation onto Tryptone Soya Agar (TSA; Oxoid, UK). A total of 88 clinical isolates of human origin (Birmingham Heartlands Hospital, UK; Aston University, UK) and 86 isolates from canine gastrointestinal infections (Veterinary Laboratories Agency, UK) were obtained. The human salmonellae were primary isolates from diarrhoeal faeces submitted to the West Midlands regional public health laboratory (now Health Protection Agency; HPA) between 1990 and 2010 as part of routine analysis for the diagnosis of gastroenteritis acquired in the UK. As such these bacterial isolates were utilised anonymously and without the requirement for specific informed consent. The corresponding Salmonella veterinary isolates of canine origin were obtained from gastrointestinal infections which were also accumulated over the same material time. Written informed consent was acquired for the retention and subsequent use of all veterinary *Salmonella* isolates. The clinical and veterinary isolates selected for inclusion in this study were therefore matched in terms of the time period over which they were isolated. In addition, where available, matched serovars were selected from the clinical culture archive with the remaining unique serovars included to balance the serovar diversity within the two populations. Cultures were incubated at 37 °C for 24 h under aerobic conditions. Table 1 lists the *Salmonella* isolates included in this study.

**Table 1:** *Salmonella* Typhimurium serovars included in the study

Canine *Salmonella enterica* Isolates			Human *Salmonella enterica* Isolates		
Serovar	ID Numbers	Percentage of total	Serovar	ID Numbers	Percentage of total
Agama	C1, C2, C67, C68	4.7	Agona	H1	1.1
Amsterdam	C3	1.2	Albuquerque	H81	1.1
Anatum	C4, C69	2.3	Anatum	H2	1.1
Arizonea	C99	1.2	Arizonea	H3	1.1
Bovismorbifican	C5	1.2	Atlanta	H4	1.1
Brandenburg	C70	1.2	Banana	H5	1.1
Carmel	C6	1.2	Bedford	H6	1.1
Cerro	C71	1.2	Berta	H7	1.1
Derby	C7, C8	2.3	Binza	H8	1.1
Dublin	C9, C10, C72, C73	4.7	Bispebjerg	H9	1.1
Durham	C11, C74	2.3	Brandenburg	H10	1.1
Enteritidis	C12, C13, C14, C15, C75	5.8	Brookfield	H11	1.1
Grumpensis	C16	1.2	Cambridge	H78	1.1
Hadar	C17	1.2	Clairbonei	H12	1.1
Havana	C18, C76	2.3	Corvallis	H13	1.1
Infantis	C19, C20, C77, C78	4.7	Driffield	H14	1.1
Isangi	C21	1.2	Ealing	H15, H16	2.3
Javiana	C22	1.2	Eastborne	H17	1.1
Kisarawe	C23	1.2	Enteritidis	H18, H19, H64, H65, H66, H83, H106, H107, H109, H110, H111	12.5
Livingstone	C24, C25, C79, C80	4.7	Ferlac	H20	1.1
London	C26, C27	2.3	Frintop	H21	1.1
Montevideo	C28, C29, C81	3.5	Havana	H77	1.1
Newport	C30, C31, C82	3.5	Heidelberg	H23	1.1
Oranienburg	C32	1.2	Infantis	H24, H108	2.3
Orion	C33	1.2	Kedougou	H25	1.1
Rissen	C83	1.2	Jejuni Penner	H88	1.1
Roodepoort	C84	1.2	Kubacha	H26	1.1
Schwarzengrund	C34, C35, C85	3.5	Lille	H46, H47, H48, H49, H50	5.7
Senftenberg	C36, C37, C86	3.5	Malawi	H27	1.1
Stourbridge	C38	1.2	Manchester	H28, H76	2.3
Telaviv	C87	1.2	Mbandaka	H30	1.1
Tennessee	C88	1.2	Maregrosso	H29	1.1
Typhimurium	C39, C40, C41, C42, C43, C44, C45, C46, C47, C48, C49, C63, C64, C65, C66, C89, C90, C91, C92, C93, C94, C95, C96, C97, C98	29.1	Montevideo	H31, H82	2.3
			Muechen	H32	1.1
			Napoli	H33	1.1
			Newport	H34	1.1
			Norwich	H35	1.1
			Panama	H36	1.1
			Pullorum	H37	1.1
			Rubislaw	H75	1.1
			Saintpaul	H38	1.1
			Santiago	H74	1.1
			Stanley	H39	1.1
			Thompson	H40, H68	2.3
			Typhimurium	H41, H42, H52, H53, H54, H55, H56, H57, H58, H59, H60, H61, H112	14.8
			Unknown	H51, H69, H70, H71, H72, H73	6.8
			Virchow	H43, H80, H103, H104	4.5
			Wraycross	H44	1.1
			Worthington	H79	1.1

### Carbon utilisation and chemical sensitivity profiling

The Biolog Gen III Microplate system was used to analyse each isolate in a series of 94 phenotypic tests including 71 carbon source utilisation assays and 23 chemical sensitivity assays providing a characteristic phenotypic profile. All nutrients and biochemicals were pre-filled and dried in the microplate wells and a tetrazolium redox dye was utilised to provide a colorimetric indication of the degree of respiration due to carbon utilisation or resistance to inhibition by chemicals. A single colony was transferred to 25 ml inoculating fluid A (IFA; Biolog Inc.) at room temperature and suspended to a cell density of 90–98% turbidity as per manufacturer’s instructions. A 150ul volume of the inoculated IFA was added to each of the 96 wells in the Microbial Identification Systems GEN III MicroPlate™ (Biolog Inc. USA) and the microplates were sealed and incubated at 37 °C for 24 h. Endpoint data of individual wells were analysed by absorbance (A) at 590 nm wavelength in a SYNERGY-HT multiwell plate reader using KC4 software (Biotek, UK). Absorbance data for each isolate were exported directly from KC4 software to Statistica version 10 software (Statsoft Inc., Tulsa, USA) for subsequent analysis.

### Minimum inhibitory concentration profiles

Antimicrobials used for the assessment of minimum inhibitory concentration (MIC) included ampicillin, chloramphenicol, tetracycline, trimethoprim, gentamycin and amoxicillin. Ampicillin, chloramphenicol, tetracycline, and trimethoprim (Sigma, UK) were dissolved in sterile distilled water (SDW) to a concentration of 256 μg/ml, while amoxicillin was reconstituted in SDW adjusted to pH 8 with 1 M ammonium hydroxide (Fisher Scientific, UK). Gentamycin was sourced at 50 mg/ml (Sigma, UK). All antimicrobials were filter sterilised using a 0.2 μm cellulose syringe filter (Nalgene, UK) and stored for a maximum of 24 h at 4 °C, prior to longer term storage at − 20 °C. MICs were determined according to the methods published by Andrews [49]. Antibiotic solutions were prepared at concentrations of 256 μg/ml; 128 μg/ml; 64 μg/ml; 32 μg/ml; 16 μg/ml; 8 μg/ml; 4 μg/ml; 2 μg/ml; 1 μg/ml; 0.5 μg/ml; 0.25 μg/ml and 0.12 μg/ml in sterile 96-well round bottom microtitre plates (Sterlin, UK). Additional dilutions of 0.06 μg/ml were prepared for trimethoprim and gentamycin.

*Salmonella* isolates were cultured on nutrient agar (NA; Oxoid, UK) at 37 °C for 24 h and a single colony was used to inoculate 10 ml nutrient both (NB; Oxoid, UK) prior to incubation at 37 °C with agitation at 200 rpm in an orbital shaking incubator (Gallenkamp, Weiss Technik UK) for 24 h. The optical density (OD) of bacterial cultures was measured at A_600nm_ and adjusted to a density of 5x10^5^cfu/ml by dilution with NB. Bacterial suspensions were combined 1:1(*v*/v) with the antibiotic preparations resulting in a final cell concentration of 2.5x10^5^cfu/ml and antibiotic exposures for each isolate at 128 μg/ml; 64 μg/ml; 32 μg/ml; 16 μg/ml; 8 μg/ml; 4 μg/ml; 2 μg/ml; 1 μg/ml; 0.5 μg/ml; 0.25 μg/ml; 0.125 μg/ml; 0.06 μg/ml and for trimethoprim and gentamycin an additional exposure concentration of 0.03 μg/ml. *Escherichia coli* strain NCTC 12241 that had a known sensitivity profile, was included as a positive control and was prepared by, and exposed to, antibiotic solutions using identical methods to the *Salmonella* isolates. Cell suspensions under exposure to antibiotics were incubated at 37 °C for 18-20 h and MIC was determined as the lowest concentration of the antimicrobial required to inhibit growth as measured by consistency of optical density at A_600nm_ at time 0 and 18 h. Published breakpoints, as provided by The European Committee on Antimicrobial Susceptibility Testing (EUCAST, 2017) for interpretation of MICs and zone diameters were used to determine resistance characteristics, while for tetracycline, similar breakpoints from the British Society for Antimicrobial Chemotherapy (BSAC, 2012) were consulted [49–51].

### Antibiotic disc susceptibility testing

Antibiotic disc susceptibility testing was performed on the *Salmonella* isolates according to standard methods published by the BSAC [52] and used to further determine the antibiotic sensitivity profiles of the isolates. *Salmonella* isolates were cultured on NA at 37 °C for 24 h and a single colony was used to inoculate 10 ml NB prior to incubation at 37 °C with agitation at 200 rpm for 24 h. Cells were harvested by centrifugation at 6000 rpm for 10 min and the supernatant was removed. The cell pellet was then suspended in 5 ml phosphate buffered saline (PBS; Sigma-Aldrich) to a turbidity of 0.5MacFarland standard [53]. A 50 μL aliquot of cell suspension was inoculated onto NA by spread plating to create a bacterial lawn and antibiotic discs (Table 2; Oxoid, UK) were applied to the surface of inoculated plates using an antibiotic disc dispenser (Thermo Scientific, UK). *E. coli* NCTC 12241 was used as a sensitive control strain and was prepared for testing by identical methods. Following incubation at 37 °C for 24 h, cultured isolates were assessed for resistance or sensitivity to each of the six antibiotics by measurement of the diameter of the zone of inhibition (mm) around the antibiotic impregnated disk according to EUCAST and BSAC standards (Table 3).

**Table 2:** Antibiotic concentration ranges for minimum inhibitory concentration and antibiotic disc sensitivity assays

Antimicrobial	Concentration range for MIC assay (μg/ml)	Disc concentration (μg/disc)
Ampicillin	0.25–128	10
Amoxicillin	0.25–128	10
Chloramphenicol	0.25–128	30
Gentamycin	0.03–128	120
Tetracycline	0.25–128	30
Trimethaprim	0.03–128	5

**Table 3:** Antibiotic breakpoints as defined by EUCAST (2017) and ^*^the British Society of Antimicrobial Chemotherapy (2012) [50–52]

Antimicrobial	MIC breakpoint (μg/ml)		Interpretation of zone diameters (mm)	
	R>	≤S	<R	S≥
Ampicillin	8	8	14	14
Amoxicillin	8	8	14	14
Chloramphenicol	8	8	17	17
Gentamycin	4	2	14	17
Tetracycline^*^	2	1	19	24
Trimethaprim	4	2	15	18

R = resistance; I = intermediate and S = sensitive

### Bacterial motility

The motility of the human and canine isolates was assessed by the ability to translocate in stab cultures of semi-solid media. Bacterial isolates were inoculated onto NA and incubated overnight at 37 °C. A single bacterial colony was inoculated into semi-solid NA (5% *w*/*v*) by vertical stab culture using an inoculation needle. Cultures were incubated at 37 °C for 24 h prior to the assessment of motility on a binary scale by visual observation of presence or absence of horizontal dispersion of growth from the vertical inoculation stab.

### Statistical analyses

#### Assessment of carbon utilisation and chemical sensitivity profiles

Student’s *t-*tests were used to compare the positive and negative assay control data between human and canine *Salmonella* isolates, using a 5% test level, to assess the appropriateness of subsequent adjustments for the controls. Absorbance values for the 71 carbon source utilisation assays were then adjusted by subtraction of the value of the negative control for each isolate and values for the 23 chemical sensitivity assays were adjusted by dividing each value by that of the positive control. Carbon utilisation and chemical sensitivity profiles of the isolates were analysed by Principal Components Analyses (PCA) to explore the correlation of variability explained with the serotype and isolate source (human or canine). Where clusters were observed, to further investigate clustering of principal components (PC) scores, isolates were separated into the clusters, and the appropriate PCA factor coordinates within each cluster were analysed by two-tailed Student’s *t-*test to investigate differences. This approach was taken in the analysis of carbon utilisation profiles between human and canine *Salmonella* isolates. A within serovar analysis was conducted on isolates of *S. enterica* serovar Typhimurium, the serovar represented in the greatest numbers, to remove serovar as a potential source of variation in carbon utilisation and chemical sensitivity profiles. Two-tailed Student’s *t*-tests were utilised to assess the degree of difference between human and canine derived isolates of *S. enterica* serovar Typhimurium. To allow for the increased likelihood of false-positives when analysing the 71 variables, *p*-values were adjusted according to the false discovery method of Benjamini and Hochberg [54] to the 5% level.

#### Antibiotic sensitivity profiles

The 174 *Salmonella* isolates were assessed for sensitivity to six antimicrobials throughout a range of concentrations to determine the MICs for growth. Kolmogorov-Smirnov two-sample tests were used to compare the distribution of MIC response variables between canine and human *Salmonella* isolates with statistical significance determined at the 5% level. The resistance/sensitivity to the same antibiotics delivered onto bacterial lawns in antibiotic impregnated discs was analysed by measurement of the zone of inhibition of bacterial growth surrounding the disc. Comparisons were similarly conducted by Kolmogorov-Smirnov two-sample tests with significance indicated at the 5% level.

## Results

### Serovar distribution

**Fig. 1:**
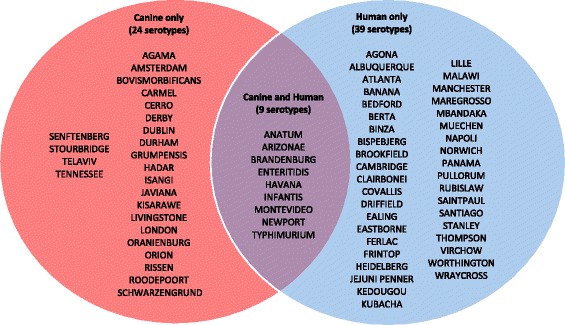
*Salmonella enterica* serovars by isolate source showing serovars isolated from humans and dogs, with those isolated from both humans and dogs at the intersection

A total of 88 human *Salmonella* isolates and 86 canine isolates were analysed for phenotypic characteristics. Within the collection 72 serovars were represented and differences in serovars were observed between isolates of human and canine origin (Fig. 1). In isolates of human origin 48 serovars were present with an additional six isolates that were of unknown serotype. Within the collection of canine isolates 33 serovars were represented with all isolates being of known serovar. Only nine serovars were shared between the collections of human and canine origin. Those shared between human and canine collections included *S. enterica* serovars Anatum, Arizonae, Brandenburg, Enteritidis, Havana, Infantis, Montevideo, Newport and Typhimurium. Of the serovars in both culture collections, many were represented by a single isolate. *S. enterica* serovar Typhimurium was the most prevalent serovar from both human and canine hosts with 13 human and 25 canine isolates. *S. enterica* serovar Enteriditis was the second most numerous serovar with 11 isolates of human origin and five of canine origin. Table 1 summarises the representation of the serovars and isolates from both human and canine hosts.

### Carbon utilisation profiles within canine and human salmonella isolates

Carbon source utilisation profiles for each isolate within the collection were assessed to further understand whether differences existed between human and canine salmonellae. Exploratory data analysis showed a largely symmetrical distribution of data points for carbon utilisation and sensitivity to inhibitory chemicals across the isolates tested. However, clusters of extreme outlying values were detected for D-galacturonic acid; γ-amino-butryric acid; mucic acid; D-serine and D-saccharic acid utilisation and for nalidixic acid sensitivity suggesting skewed or bimodal distributions of utilisation of, or of sensitivity to, these chemicals within the salmonellae tested. Growth with tetrazolium violet and tetrazolium blue were considered experimental controls demonstrating resistance to the redox dye utilised within the Biolog system and were close to, or at the limit of detection. Consequently they were excluded from subsequent multivariate analysis.

Student’s *t-*tests suggested no significant difference existed for the positive (*p* = 0.849) or negative (*p* = 0.984) assay controls between the canine (mean ± SE: 2.157 ± 0.216 and 0.382 ± 0.101, respectively) and human *Salmonella* isolates (mean ± SE: 2.198 ± 0.220 and 0.393 ± 0.101, respectively). Consequently, adjustment of the carbon utilisation values using the negative control and chemical sensitivity values using the positive control values was considered to be valid.

**Fig. 2:**
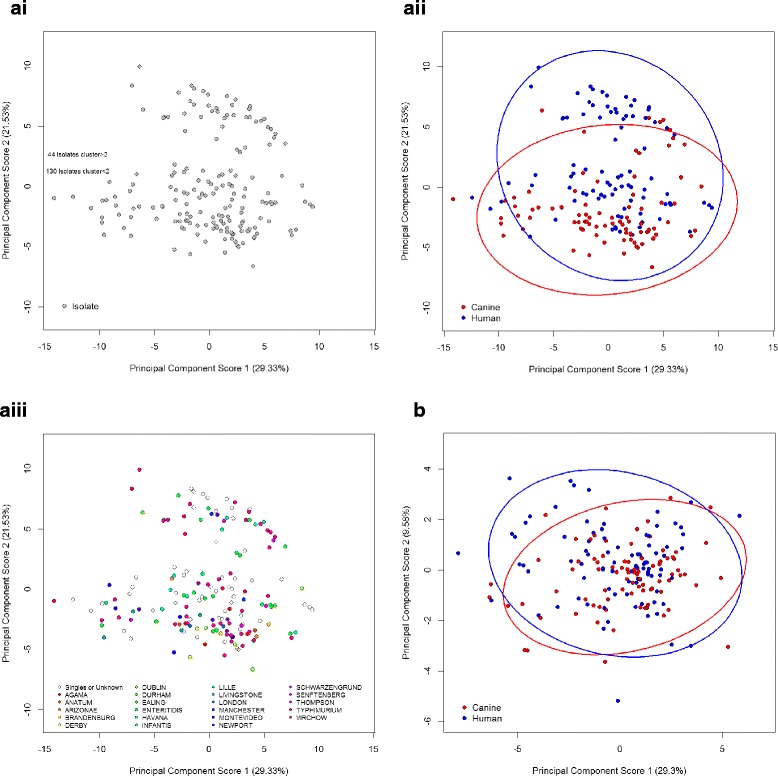
Scatterplot of principal component scores 1 and 2 from analysis of (**a**) carbon utilisation profiles, (i) demonstrating apparent cluster line and coloured by (ii) host species and (iii) isolate serovar and (**b**) chemical sensitivity profiles. The 95% confidence ellipses for the scores are shown with the corresponding colour

Analysis of carbon utilisation profiles by PCA demonstrated that the first two factors explained approximately 50% of the total variability in the data with factor 1 explaining 29.33% and factor 2 explaining 21.53% of the inherent variability. Projection of the isolates onto the plane defined by the first two factors revealed two apparent clusters of isolates, chiefly across the range of factor 2 (Fig. 2a). One cluster contained 130 isolates with factor 2 values below 2.0, the other cluster contained 44 isolates with factor 2 values above 2.0 (Fig. 2ai). The lower cluster of 130 isolates comprised 42% human and 58% canine isolates, while the upper cluster of 44 isolates was composed of 75% human and 25% canine isolates. Although clusters did not coincide with the source of the isolates, i.e. with human and canine host species, the canine isolates within each cluster appeared to have lower factor 2 values (Fig. 2aii). Two-tailed Student’s *t-*test confirmed a significant difference between the factor 2 values of human and canine *Salmonella* isolates within the upper cluster (*p* = 0.0001) and the lower cluster (*p* = < 0.0001). When serotype was overlaid onto the scatterplot, no determinable pattern in carbon utilisation profiles with serotype was detected (Fig. 2aiii).

Correlations between Factor 2 and the carbon utilisation values were ranked by absolute R value to identify the phenotypes most highly correlated with the separation of the two clusters (Table 4). The clustering observed within the carbon utilisation dataset across factor 2, was analysed further by ranking of the carbon sources according to influence on the degree of separation between the human and canine samples. The difference in the means ranged between 0.26 for hydroxy-phenylacetic acid and − 0.63 for myoinositol (Table 5). After adjustment for false discovery rate (FDR), two-tailed paired Student’s *t-*tests suggested that significant differences in the utilisation of 33 carbon sources existed between human and canine derived *S. enterica* Typhimurium isolates.

**Table 4:** Carbon utilisation variables across all 174 *Salmonella* isolates correlated with principal component factor 2 ordered by absolute R value

Rank in correlation with Factor 2	Carbon Source	PCA Loadings Factor 2
1	α–D-Glucose	0.834
2	D-Maltose	0.802
3	D-Raffinose	0.720
4	D-Melibiose	0.720
5	D-Cellobiose	0.709
6	Glycerol	0.692
7	D-Mannitol	0.690
8	N-Acetyl D-Glucosamine	0.681
9	D-Fructose	0.677
10	L-Fucose	0.659
11	α-Hydroxy Butyric Acid	−0.657
12	β-Methyl D-Glucoside	0.651
13	Sucrose	0.639
14	D-Arabitol	0.624
15	α-Keto Butyric Acid	−0.598
16	Bromo Succinic Acid	−0.583
17	α-D-Lactose	0.572
18	Propionic Acid	−0.570
19	L-Glutamic Acid	−0.563
20	Formic Acid	−0.560
21	D-Galactose	0.554
22	D-Turanose	0.546
23	L-Histidine	−0.545
24	D-Fucose	0.542
25	Myoinositol	0.533
26	Gentiobiose	0.526
27	Tween 40	−0.514
28	N-AcetylDGalactosamine	0.501

Table 4 ranks the carbon sources by their factor loadings in PCA factor 2. The largest proportion of carbon sources highly correlated with factor 2 were simple sugars including monomers, disaccharides and trisaccharides containing glucose including α–D-glucose, D-maltose, D-raffinose, D-melibiose and D-cellobiose

**Table 5:** Carbon utilisation by *Salmonella* Typhimurium isolates ranked by degree of separation between human and canine derived organisms, where statistically significant according to paired Student’s *t*-tests after adjustment for FDR

Carbon source	Mean		Std. Dev.		Difference in the means	*t*-test	
	Human	Canine	Human	Canine		*t*-value	FDR *p*-value
Gentiobiose	−0.181	0.086	0.071	0.129	−0.267	−6.904	< 0.001
D-Mannitol	−0.024	0.273	0.065	0.169	−0.297	−6.088	< 0.001
Myo-inositol	−0.087	0.545	0.109	0.383	−0.632	−5.800	< 0.001
N-Acetyl-D-Galactosamine	−0.127	0.113	0.082	0.139	−0.24	−5.695	< 0.001
α-D-Lactose	−0.127	0.075	0.058	0.121	−0.202	−5.665	< 0.001
D-Fructose	0.068	0.454	0.113	0.258	−0.386	−5.115	< 0.001
α-Hydroxy-Butyric Acid	0.526	0.348	0.116	0.095	0.178	5.082	< 0.001
D-Maltose	0.11	0.401	0.142	0.204	−0.291	−4.569	0.001
α–D-Glucose	−0.039	0.175	0.127	0.144	−0.214	−4.515	0.001
D-Melibiose	0.118	0.394	0.122	0.209	−0.276	−4.368	0.001
D-Aspartic Acid	0.733	0.483	0.135	0.181	0.25	4.359	0.001
L-Glutamic Acid	0.578	0.398	0.128	0.121	0.18	4.253	0.001
Bromo-Succinic Acid	0.602	0.424	0.158	0.109	0.178	4.074	0.002
L-Fucose	0.288	0.686	0.174	0.361	−0.398	−3.747	0.004
L-Histidine	0.449	0.276	0.148	0.134	0.173	3.655	0.005
Tween 40	0.477	0.318	0.135	0.125	0.159	3.631	0.005
‘Hydroxy-Phenylacetic Acid	0.976	0.715	0.183	0.23	0.261	3.539	0.006
Glycyl-Proline	0.748	0.575	0.133	0.149	0.173	3.516	0.006
Propionic Acid	0.684	0.497	0.149	0.163	0.187	3.457	0.007
D-Fucose	−0.057	0.083	0.09	0.131	−0.14	−3.441	0.007
D-Arabitol	−0.046	0.099	0.107	0.133	−0.145	−3.391	0.007
D-Galactose	0.322	0.609	0.118	0.296	−0.287	−3.337	0.008
D-Cellobiose	−0.056	0.071	0.105	0.117	−0.127	−3.295	0.009
Formic Acid	0.352	0.211	0.124	0.131	0.141	3.215	0.010
D-Sorbitol	0.23	0.367	0.135	0.119	−0.137	−3.207	0.010
Rifamycin SV	1.056	0.965	0.103	0.074	0.091	3.148	0.012
D-Raffinose	−0.001	0.161	0.186	0.136	−0.162	−3.065	0.014
Sucrose	−0.072	0.03	0.095	0.1	−0.102	−3.034	0.015
Glycerol	0.551	0.846	0.287	0.314	−0.295	−2.826	0.024
D-Turanose	−0.047	0.046	0.09	0.098	−0.093	−2.824	0.024
N-Acetyl-D-Glucosamine	0.147	0.527	0.132	0.501	−0.38	−2.670	0.034
Acetic Acid	0.683	0.526	0.166	0.179	0.157	2.628	0.036
Gelatin	0.077	0.029	0.061	0.052	0.048	2.539	0.043
α-Keto-Butyric Acid	0.411	0.317	0.115	0.109	0.094	2.481	0.048

Table 5 ranks carbon sources by the value of significance of difference in the means between human and canine derived *Salmonella* isolates. Simple sugars are highly represented within the ten highest ranking compounds including significant differences in gentiobiose, α-D-lactose, D-fructose, D-maltose, α–D-glucose and D-melibiose utilisation. With the exception of D-fructose and α-hydroxybutyric acid these simple sugars were generally utilised to a greater extent by isolates of canine origin rather than human *Salmonella* isolates

### Chemical sensitivity profiles within canine and human salmonella isolates

PCA of data from the 23 chemical sensitivity tests across the 174 human and canine *Salmonella* isolates demonstrated that the first component explained 29.30% of the variability, while the second explained 9.58% of the variability. Projection onto a scatterplot according to principal components 1 and 2 gave no indication of clustering of the isolates based on their chemical sensitivity profiles (Fig. 2b). Two-tailed paired Student’s *t-*tests suggested that a significant difference in the response to a single chemical (Rifamycin SV) existed between human and canine derived *S. enterica* Typhimurium isolates.

### Within Serovar assessment of human and canine salmonella enterica typhimurium profiles

**Fig. 3:**
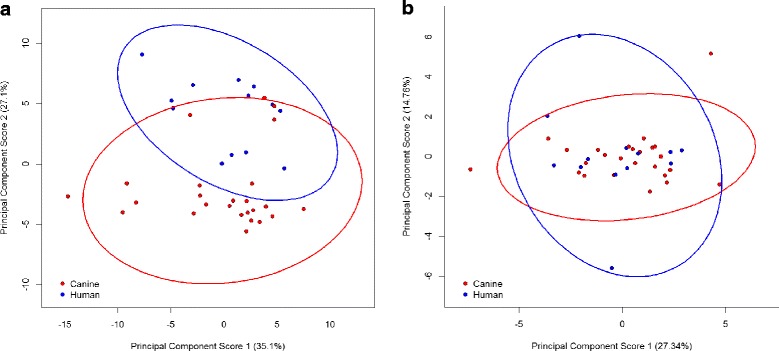
Principal component scores 1 and 2 from analysis of (**a**) carbon utilisation profiles and (**b**) chemical sensitivity profiles of *S. enterica* serovar Typhimurium isolates from humans (blue) and dogs (red), with 95% confidence ellipses

In order to further understand the degree of difference in carbon utilisation profiles of human and canine salmonellae, and determine whether differences in serotype distribution contributed to observed differences in carbon source utilisation, analysis of a single serovar was conducted. This aimed to eliminate variation between serovars as a potential confounding factor. As the most represented serovar within the culture collection, *S. enterica* serovar Typhimurium was utilised in the analyses to assess redox data across the 71 carbon sources and 23 chemical inhibition assays produced by 13 human and 25 canine *Salmonella* isolates. PCA of the carbon utilisation values from the 38 *S. enterica* Typhimurium isolates tested, demonstrated that the first component explained 35.10% of the variability and the second component explained 27.10% of the variability. Clustering of human and canine isolates was observed across factor 2 with the majority (21 of 25) of canine species falling into a cluster with factor 2 values below − 1.0 and with all 13 human isolates within a cluster with factor 2 values greater than − 1.0 (Fig. 3a). Ranking of the carbon sources by PCA factor 2 coordinates (absolute values) indicated those most highly correlated with the separation of isolates across factor 2 and demonstrated identical rankings to the analysis across all serovars in Table 4 (data not shown).

Similarly to the cross serotype analysis, PCA of the chemical sensitivity values from the 38 *S. enterica* Typhimurium isolates demonstrated no separation in the data across either factor with a single cluster and small number of outlying isolates (Fig. 3b).

### Antibiotic minimum inhibitory concentration profiles

Assessment of the distribution of response variables between the human and canine isolates using Kolmogorov-Smirnov two-sample tests for each individual antibiotic, suggested significant differences in the MIC profiles of ampicillin, amoxicillin and chloramphenicol between isolates derived from human and canine infections (Table 6). No significant difference was observed in the distribution of sensitivity to gentamycin, tetracycline and trimethoprim between human and canine isolates. The sensitivity of *Salmonella* isolates to four of the six antimicrobials tested (ampicillin, chloramphenicol, gentamycin and trimethoprim) showed a normal distribution of minimum growth-inhibitory concentrations across the collection of 174 *Salmonella* isolates (Table 6). For both ampicillin and chloramphenicol MIC responses, canine isolates were over-represented at the highest MIC compared to their human counterparts. Organisms with a MIC_ampicillin_ at the upper limit of testing (128 μg/ml) or above represented 10% of the human *Salmonella* isolates tested and 30% of those of canine origin. Similarly, sensitivity to chloramphenicol at the upper limit of testing or above was identified in 6.3% of the human salmonellae and 20.9% of those isolated from dogs. A bimodal response in the sensitivity of the isolates to amoxicillin was observed with peaks in MIC response at 1 μg/ml and 8 μg/ml. When considered in terms of isolate source an MIC_amoxicillin_ at or above the upper limit of testing (≤128 μg/ml) was found in 26.7% of the canine isolates and 6.3% of salmonellae of human origin. A skewed distribution of sensitivity to tetracycline was identified across the culture collection and in both human and canine sub-collections peaking at a MIC of 1 μg/ml.

**Table 6:** Antibiotic minimum inhibitory concentration response distributions by antibiotic compound across all isolates and by *Salmonella* source, with Kolmgorov-Smirnov (K-S) test results

MIC	Ampicillin			Amoxicillin			Chloramphenicol			Gentamicin			Tetracycline			Trimethoprim		
	All Isolates	Human	Canine	All Isolates	Human	Canine	All Isolates	Human	Canine	All Isolates	Human	Canine	All Isolates	Human	Canine	All Isolates	Human	Canine
< 0.25							1	1		1		1						
0.25	1		1										1		1			
0.5	6	2	4	21	12	9	1		1	4	3	1	11	6	5	4	3	1
1	11	2	9	36	18	18	6	3	3	23	13	10	49	27	22	9	8	1
2	24	15	9	25	10	15	19	11	8	20	10	10	39	19	20	26	13	13
4	12	4	8	6	5	1	28	16	12	87	47	40	18	4	14	95	47	48
8	43	22	21	35	23	12	65	31	34	3		3	26	12	14	7	1	6
16	16	14	2	5	3	2	7	7		4	3	1	10	4	6	6	2	4
32	17	13	4	6	4	2	7	6	1	12	2	10	3	2	1	1	1	
64	2		2	4		4	9		9	1		1	6	4	2	8	1	7
128	17	8	9	12	5	7	12	5	7	5	2	3	3	2	1	7	3	4
> 128	17		17	16		16	11		11	6		6				3	1	2
K-S		d = 0.2256			d = 0.2515			d = 0.2515			d = 0.1916			d = 0.0919			d = 0.1549	
		*p* < 0.05			*p* < 0.025			*p* < 0.025			*p* < 0.1			*p* > 0.1			*p* > 0.1	

### Antibiotic disc susceptibility testing

Variation also existed across the 174 isolates with regards to their sensitivity to antibiotics, applied to inoculated agar by diffusion from ampicillin, amoxicillin, chloramphenicol, gentamycin, tetracycline and trimethoprim impregnated discs. Comparison of the sensitivity responses of isolates by source (human or canine) using Kolmogorov-Smirnov two sample *t-*tests suggested no difference in the response to antibiotic discs between isolates of human and canine origin (*p* > 0.1 for all of the antibiotics tested).

### Bacterial motility

All of the canine and human isolates were motile as demonstrated by horizontal translocation of bacterial growth from vertical stab cultures in semi-solid growth media.

## Discussion

This research describes a detailed phenotypic profiling of *Salmonella enterica* isolates from clinical enteric infections of humans and dogs. It should be noted that the study does not represent an epidemiological analysis. The isolates and serovars selected for analysis were simply representative of those present in the culture collections sampled and not of a defined population or area. Furthermore these findings relate to pathogenic salmonellae, since culture collections contained clinical isolates associated with disease in either humans or dogs. In dogs those associated with clinical infections may represent only a proportion of salmonellae, since carriage without clinical signs of infection and sub-clinical shedding is also reported to varying extents [2, 10, 11, 55]. In fact, the prevalence of *Salmonella* in healthy dogs and cats in some populations may be similar to that in diarrheic animals [56, 57] with incidence rates reportedly differing across geographies [57, 58]. In UK populations however, such as that in which the study culture collections were obtained, previous studies describe very low carriage rates in healthy dogs [12, 14]. Since clinical outcome of infection is considered to be highly dependent on strain for *S. enterica* isolates [2], the findings of this study are more likely to represent the general canine *Salmonella* population of the region (UK) than similar studies conducted in regions with higher rates of carriage reported without clinical signs.

It remains unclear as to whether this population of salmonellae reflects that of the general population, or is a characteristic of the collection obtained for study. However, despite these restrictions, the serotypes observed within the culture collections from humans and dogs in this UK study are suggestive of differences in the serovars isolated from clinical *Salmonella* infections in these hosts. The serovars represented in the canine *Salmonella* collection of this study are similar to those identified in a recent study of 442 salmonellae isolated from dogs in the UK between 1957 and 2012 [9]. Philbey et al. reported that the most prevalent serotypes represented in dogs were *S. enterica* Typhimurium (44.3%, 196 isolates); Dublin (9.0%, 40 isolates); Enteritidis (6.3%, 28 isolates; 6.3%) and Montevideo (4.3%, 19 isolates) [9]. These serovars were also present in the canine collection of the reported study with serovars Typhimurium (29%) and Enteritidis (5.8%) the most numerous and also present in the human collection along with serovar Montevideo (3.5% of canine isolates), while serovar Dublin (4.7%) was present only in the collection of isolates cultured from dogs. Based on the serotypes, phage types and antibiotic sensitivity profiles detected the authors concluded that the UK canine population encounters a range of *S. enterica* serovars and as such pose a zoonotic risk.

Detailed characterisation of the isolates by phenotypic analysis of carbon utilisation profiles, supported the hypothesis that differences exist in canine and human salmonellae. More than 50% of the variation in carbon utilisation profiles between the 174 salmonellae analysed was explained by the first two factors, with projection of the isolates plotted against these two factors resulting in two clusters. Within the clusters differential representation of isolate host species was observed. Carbon utilisation profiles were not observed to cluster with serotype, however given the large number of singlet serotype representatives, interpretation of the analysis by serotype proved difficult. When considering serovars containing numerous isolates, isolates from *S. enterica* serovars Typhimurium and Enterica were distributed throughout the upper and lower clusters and across PCA factor 2 the main separator of canine and human salmonellae. Once the presence of two clusters were accounted for, a significant difference was identified in the carbon utilisation profiles from human and canine sources with canine isolates showing generally lower factor 2 values. This suggested that the utilisation of carbon sources contributing most strongly to PCA factor 2 may best distinguish between human and canine host species. This separation, both in the clusters and between human and canine isolation source was supported by a within serovar analysis in which isolates of *S. enterica* Typhimurium, the most numerous serovar, were assessed by PCA.

Analysis of correlations between carbon source utilisation and factor 2 was used to examine the most influential carbon sources in the separation across factor 2. The largest proportion of carbon sources highly correlated with factor 2, were simple sugars including monomers, disaccharides and trisaccharides containing glucose including α–D-glucose, D-maltose, D-raffinose, D-melibiose and D-cellobiose. These sources were more highly correlated with the lower cluster across factor 2 comprising 42% human and 58% canine *Salmonella* isolates. Carbon sources most highly correlated with the upper cluster across factor 2 in which human isolates were comparatively over-represented (ie. comprising 75% human and 25% canine isolates) included α-hydroxybutyric acid, α-ketobutyric acid involved in amino acid metabolism and L-histidine. Based on the comparatively restricted levels of simple sugars available within pet foods and higher protein content compared to human dietary intakes, the observed difference in carbon utilisation would not be expected to be a result of nutritional adaptation, unless differences in the digestive processes between hosts create disparity in the digesta at the site of colonisation. When carbon source utilisation data from isolates of *S. enterica* serovar Typhimurium only were ranked by their PCA factor 2 coordinates to determine the strength of association with the clusters and isolate source, the ranking and direction of correlation mirrored that of the total culture collection suggesting that differences in serovar across the human and canine isolate collections did not influence the clustering within carbon utilisation characteristics.

Student’s *t-*tests used to assess differences in the means of carbon source utilisation and chemical sensitivities for human and canine isolates demonstrated significant differences in 34 of the variables after adjustment for FDR. Only one of these represented a chemical sensitivity assay, which was to the antibiotic rifamycin. Growth on exposure to this antimicrobial was significantly higher in isolates of human origin than those isolated from dogs. Greater sensitivity to the compound in canine isolates is perhaps not surprising, given the propensity for rapid development of resistance to rifampicin and the lack of approval of rifampicin for veterinary use. As in the correlations with PCA factor 2, rankings by significance of differences in the means between human and canine isolates resulted in simple sugars being highly represented within the ten highest ranking compounds. These included significant differences in growth on gentiobiose, α-D-lactose, D-fructose, D-maltose, α–D-glucose and D-melibiose. These simple sugars were generally utilised to a greater extent by isolates of canine origin rather than human *Salmonella* isolates with the exception of D-fructose and α-hydroxybutyric acid.

The divergence identified within the carbon utilisation profiles could reflect direct adaptation to nutritional differences in the host. The extent to which host nutrition and digesta characteristics influence the microbiota is unclear, particularly between similarly omnivorous mammals such as the human and the dog. Host immune factors, the co-residing microbiota, prior clinical interventions and environmental exposures to *Salmonella* spp. linked to behavioural and dietary sources all have the potential to influence the reservoir of salmonellae inhabiting the host. The observed differences in carbon source utilisation of canine and human isolates are nevertheless suggestive of separation of isolates from humans and dogs, and may simply represent an indirect consequence of the evolutionary separation of the organisms infecting humans and dogs. This would be suggestive of distinction in the reservoirs for human and canine clinical isolates, since shared reservoirs of infection would dilute evolutionary divergence in carbon utilisation profiles. The direction of the differences in the mean utilisation of carbon sources most influential on PCA factor 2 may also point towards an evolutionary divergence in distinct reservoirs of human and canine salmonellae. Since canine diets possess lower levels of simple sugars than those typical of humans, direct adaptation of canine *Salmonella* isolates to the nutritional intake of the dog would be considered unlikely to direct organisms to more effectively utilise these dietary components. Particularly given that protein and complex carbohydrates represent the predominant energy sources in commercial dog foods.

Variation in chemical sensitivity profiles reflecting the degree of resistance of isolates to 23 different chemicals was relatively consistent across isolates from both hosts. Almost 40% of the variability in PCA scores was explained by factors 1 and 2, however patterns in the distribution or clustering of isolates were not observed across either factor based on chemical sensitivity profiles. These findings were consistent in PCA across all serovars as well as in that considering only *S. enterica* Typhimurium. The results of *t-*test’s for comparison of differences in mean values of carbon source utilisation and chemical sensitivities between human and canine isolates also supported this, with significant differences (*p* = < 0.05) observed in mean growth on 33 carbon sources but only a single inhibitory chemical. Hence, chemical sensitivity profiling was not useful in distinguishing between isolate source.

Assessment of antibiotic resistance as measured by diffusion through solid growth media from antibiotic impregnated discs similarly did not support the detection of differences in sensitivity of human and canine isolates to ampicillin, amoxicillin, chloramphenicol, gentamycin, tetracycline or trimethroprim. However, differences in the MIC profiles in salmonellae of human and canine origin were observed in more detailed analyses of sensitivity distribution across the concentration range tested for ampicillin, amoxicillin and chloramphenicol. For these antibiotics the MIC profiles of canine isolates were generally skewed towards higher concentrations within the range tested compared to salmonellae of human origin. No differences in MIC distribution profiles of human and canine isolates were observed on exposure to gentamycin, tetracycline or trimethroprim.

The disc diffusion antimicrobial sensitivity methods appeared to lack the sensitivity required to detect differences in susceptibility of isolates to ampicillin, chloramphenicol and amoxicillin. The antimicrobial concentrations delivered on impregnated discs was in the mid-range of the concentrations used for MIC_ampicilin_ and MIC_amoxicillin_ analysis and around the upper-quartile of the concentration range for MIC_chloramphenicol_. Hence, single point antimicrobial sensitivity assays did not possess the discriminatory power of MIC profiles for the comparison of antibiotic sensitivity in human and canine salmonellae.

The differences detected in antibiotic sensitivity profiles may reflect the greater prevalence of antibiotic usage in veterinary medicine, particularly since ampicillin, amoxicillin and chloramphenicol represent antibiotics used commonly in veterinary medicine [59–61]. Increased resistance in canine isolates across three of the six antimicrobial compounds tested compared to human isolates may be suggestive of multiple or chronic exposure to antibiotic compounds leading to an accumulation of resistance within canine salmonellae. Despite intentional reductions in the use of antibiotics in human medicine over recent decades, appropriate antibiotic usage by clinicians remains a focus for veterinary associations such as the World Small Animal Veterinary Association (WSAVA). The impact of veterinary antibiotic usage has been noted in other species, and could be compounded by current pet feeding trends. It is of note that a study of faecal *E. coli* isolated from UK dogs identified recent use of antimicrobials and being fed raw poultry as risk factors in the carriage of multidrug resistant (defined as resistance to three or more antimicrobial classes) isolates [62].

Taken together, differences observed in the carbon utilisation profiles and antibiotic sensitivity profiles of canine and human salmonellae are indicative that, at least for clinical isolates causing enteritis in their host, there is a separation in the reservoirs of salmonellae infecting dogs and humans. Similar findings were reported by Mathers et al. who, using whole genome sequencing and drug resistance phenotypes to understand the detailed phylogenetic relationships within a UK national collection of isolates from humans and animals, surmised that *Salmonella* isolates are largely maintained within separate human and animal populations [34, 63]. These detailed genetic analyses provide still further degrees of resolution beyond those possible by detailed carbon utilisation profiles, yet these results are in agreement suggesting studies using levels of scrutiny beyond sero/phage-typing and antibiotic sensitivity profiles are perhaps required to appreciate the full extent of the microbial ecology and transmission routes in salmonellosis.

Given the multiple clinical outcomes of *Salmonella* carriage in dogs, it would be of interest to determine the serotypes, carbon utilisation and MIC profiles of salmonellae from a-symptomatic dogs and compare these to those profiles associated with disease in humans and dogs. Comparison of carbon utilisation and antibiotic MIC distribution profiles in isolates from these cohorts may enhance the understanding of pathogenicity determinants and support studies to understand the source of isolates associated with clinical and sub-clinical infections. Though the apparently low sub-clinical carriage rates for *Salmonella* in UK dogs may introduce inherent challenges in such research.

## Conclusions

Taken together these findings suggest that the analysis of carbon source utilisation profiles and MIC distribution profiles represent useful approaches to determine differences between pathogenic salmonellae isolated from humans and dogs. Distinct carbon utilisation profiles and distributions in MIC ranges of three commonly used antibiotics were observed in salmonellae from humans and dogs. A higher level of resistance to ampicillin, amoxicillin and chloramphenicol was detected in canine salmonellae pointing to the relatively higher antibiotic usage in veterinary medicine compared to treatments in human medicine. The dissimilarity in phenotypic characteristics is suggestive of separation in the reservoirs of salmonellae causing clinical infections in humans and dogs.

## Abbreviations

BBSRC: Biotechnology & Biological Sciences Research Council
BSAC: British Society for Antimicrobial Chemotherapy
cfu: colony forming units
*E. coli*: *Escherichia coli*
EUCAST: The European Committee on Antimicrobial Susceptibility Testing
FDR: false discovery rate
FoodNet: The Foodborne Diseases Active Surveillance Network
HPA: Health Protection Agency
IFA: Inoculating fluid A
MIC: minimum inhibitory concentration
NA: nutrient agar
NB: nutrient both
OD: optical density
PBS: phosphate buffered saline
PC: principal components
PCA: Principal Components Analyses
rpm: revolutions per minute
*S. enterica*: *Salmonella enterica*
SDW: sterile distilled water
TSA: Tryptone Soya Agar
*v*/v: volume to volume
*w*/*v*: weight to volume
WSAVA: World Small Animal Veterinary Association
